# Successful closure of the ventricular septal defect;A rare complication after transcatheter aortic valve replacement

**DOI:** 10.34172/jcvtr.2022.27

**Published:** 2022-09-10

**Authors:** Saadet Demirtas Inci, Murat Tulmaç, Cagatay Tunca, Tolgahan Efe, Hakan Güllü

**Affiliations:** Health Sciences University Yildirim Beyazit Diskapi Education And Research Hospital, Cardiology Department, Ankara, Turkey

**Keywords:** Transcatheter Aortic Valve Replacement, Ventricular Septal Defect, Aortic Valve Stenosis

## Abstract

In this report, we present a patient with ventricular septal defect (VSD) that was detected at follow-up one month after transcatheter aortic valve implantation (TAVI) and successfully closed percutaneously. Before the procedure, a 29 mm Portico self-expanding aortic valve prosthesis was placed in the heavy calcific aortic valve position, and then the balloon was dilated due to aortic insufficiency and excellent results were obtained. One month after TAVI, the patient complained of shortness of breath at rest, and on physical examination a pansystolic murmur was detected. Transthoracic echocardiography (TTE) revealed a well-functioning prosthetic aortic valve; however, a VSD was detected causing left-to-right shunt in the interventricular septum. Later, we performed the interventional treatment of the defect using the Amplatzer muscular VSD occluder device with the transfemoral approach. Currently, five months after the combined procedure, the patient showed a significant improvement in symptoms and no significant shunt was observed.

## Introduction

 Transcatheter aortic valve implantation (TAVI) is the preferred method in symptomatic patients with severe aortic valve stenosis who are at moderate to high risk for traditional surgery due to age and other comorbidities.^1,2^ Ventricular septal defect (VSD) is an extremely rare but serious complication of TAVI. Mechanisms and risk factors for this complication are not well understood. As the number of the TAVI procedure that occurs increases, the encounter with this complication increases.

 We present a case of a TAVI procedure using a 29 mm Portico self-expandible valve prosthesis with severe calcific aortic stenosis causing an iatrogenic perimembranous VSD thought to be caused by a calcific nodule located in the left ventricular outflow tract (LVOT). The defect was successfully closed through percutaneous technique using three-dimensional (3D) transesophageal echocardiography (TOE) with the Amplatzer muscular VSD occlude (St. Jude Medical, MN) in another session.

## Case Presentation

**Figure 1:**
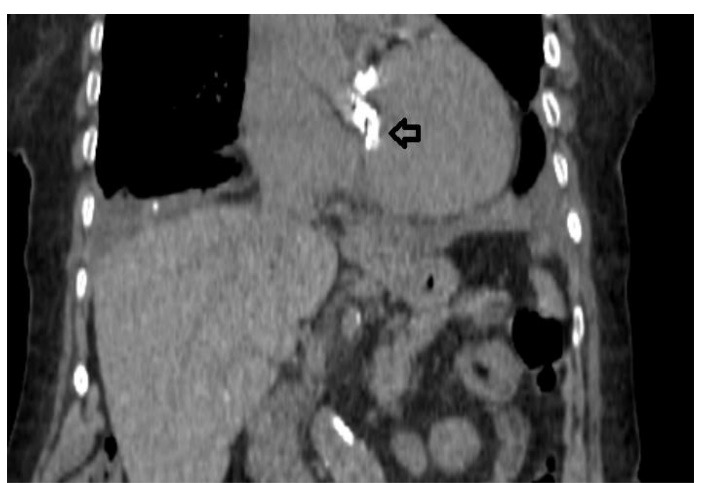
Calcific nodule image in the left ventricular outflow tract in multislice cardiac computed tomography (arrow).

**Figure 2:**
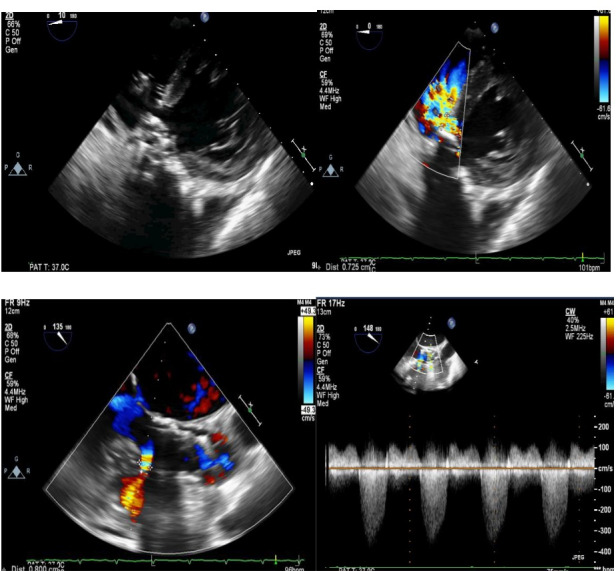
Ventricular septal defect image after TAVI in transesophageal echocardiography.

**Figure 3:**
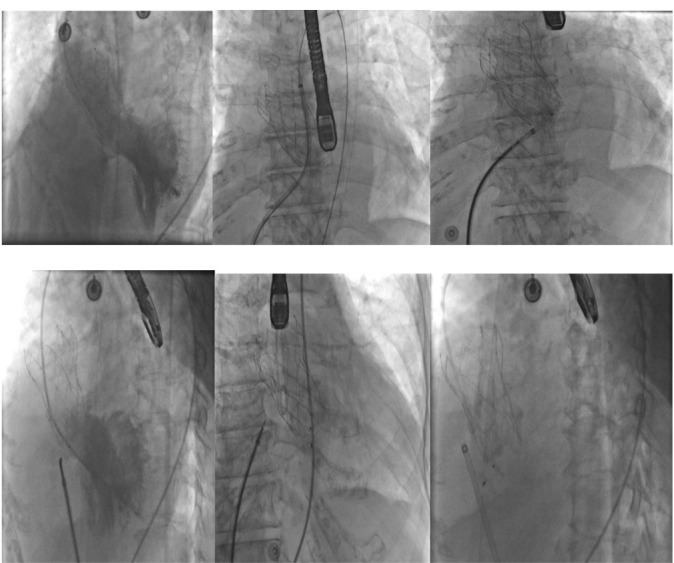
Angiographic view of the percutaneous closure of the ventricular septal defect.

**Figure 4:**
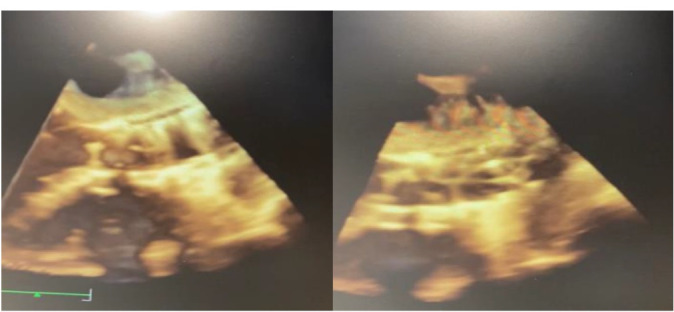
3D Transesophageal view of the ventricular septal defect in the closing process.

**Figure 5:**
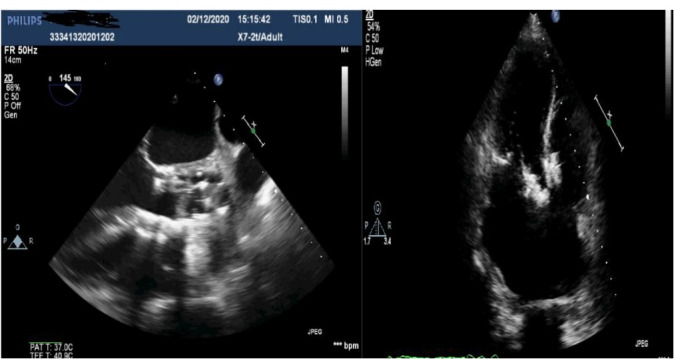
Transesophageal and transthoracic images of the VSD muscular device after the closure of the ventricular septal defect.

 The patient was an active 82-year-old woman (NYHA IV) with shortness of breath at rest. Her comorbidities included hypertension and asthma history. During her examination, she was found to have normal left ventricular (LV) systolic function (ejection fraction; 60%) and severe calcific aortic valve stenosis (aortic valve area: 0.7 cm^2^; peak / mean gradient: 85/55 mm Hg) on transthoracic echocardiography (TTE). Coronary angiography was found to have normal coronary vessels. The patient with 5.1% STS score was decided to undergo TAVI by our Heart Team. On multi-slice cardiac computed tomography (CT), severe calcification from the aortic valve level and calcified nodule towards the LVOT were noted. Calcific nodule extending to LVOT shown in Figure 1. The procedure was briefly performed as follows: A 6-F sheath was placed in the right common femoral artery and vein then proglide was inserted. A stiff wire was then inserted through a pig tail catheter. 19 F delivery catheter has been advanced from descending aorta. By performing fast pacing, a nucleus 20×40 mm balloon was predilated and a self-expandable 29 mm Portico prosthesis was implanted. At this stage, there was second grade aortic ınsufficiency (AR) and the prosthesis was postdilated with nucleus 25×40 mm balloon. After the procedure, a very good result was obtained with only minimal insufficiency and elimination of the aortic gradient. No problem developed during the follow-up during hospitalization and no obvious pathology was detected in the post-procedure follow-up echocardiography. However, a pansystolic murmur was detected in the physical examination of the patient with dyspnea at the follow-up 1 month later, and aortic Portico prosthesis (maximum gradient:11 mm Hg, minimal insufficiency) was detected on TTE. However, the right chambers were dilated with significant tricuspid regurgitation and an increased systolic pulmonary artery pressure of 60 mm Hg. In addition, a perimembranous VSD 7 mm was evident beginning from the edge of the prostetic valve, resulting in a significant left-to-right shunt. Based on these findings Figure 2, it was decided to perform interventional treatment of the defect using the Amplatzer muscular VSD occluder. Right femoral artery and vein canulated using 6-F sheath. A pigtail catheter was passed to the LV. A 0.035 hydrophilic wire and 5-F JR-3.5 catheter were then passed through the VSD to the RV. The wire unintentionally went into the inferior vena cava and the catheter sent over the wire to the inferior vena cava. Then the hidrofilic wire was exchanged with a 0.035 300 cm Noodle wire. The wire was snared in the inferior vena cava and externalized through the right femoral vein. From the right femoral vein a 7-f delivery sheath sent to the ascending aorta through inferior vena cava and the VSD. VSD diameter was measured 7 mm by ventriculography and 8 mm with TOE. Later, 10 mm Amplatzer muscular VSD occluder device was sent through the long sheath, and the first disc was opened in the ascending aorta. Then the system was pulled back to the VSD and the second disc was unsheated (Figure 3). On 3D-TOE the occluder device was in a correct position, and there was a minimal shunt possibly through the occluder device (Figure 4). It was disconnected from the delivery cable after confirming that there was no compression or problem with the aortic valve. Two days after the procedure, the device was in a good position, there was mild tricuspid regurgitation and systolic pulmonary artery pressure was 40 mmHg (Figure 5). After one week of follow-up, the patient was discharged home. It continues to be active with significant improvement in shortness of breath (NYHA I-II) twelve months after the procedure.

## Discussion

 Various serious complications may develop during TAVI. VSD development is a rare and potentially fatal complication. Some publications report that VSD is seen at a rate of 1.5%.^3-5^ Although the risk factors for developing VSD during TAVI are not fully known, several factors are thought to cause it. In general, several patient-related factors, such as the severity, location, or asymmetric distribution of calcification, and ventricular morphology, or procedural factors such as excessive balloon expansion before or after valve insertion, and / or higher placement of the valve are thought to cause VSD after TAVI. That is, iatrogenic VSD following the TAVI procedure can take two forms: (1) dilatation of the calcific aortic valve before or after the procedure may result in damage of calcification to the interventricular septum, and/or (2) there may be direct damage to the interventricular septum during valve dilatation. 1 degree relative to AR at the end of TAVI is quite common; If AR is more than 2 degree, it is not accepted. In general, one of the causes of AR after TAVI is incomplete expansion of the prosthesis,^6^ and in such cases postdilatation greatly improves the outcome. In our case, balloon dilatation was not performed before the valve insertion, but balloon dilatation was performed after valve insertion due to the detection of second degree AR after the procedure. Before the procedure, our patient had severe calcific extentions into the LVOT on CT, and we think that the calcific nodule detected in this outflow tract caused the development of VSD. Approximately, Twenty iatrogenic VSD cases have been reported in the literature after TAVI. As in our case, it has been reported that membranous and perimembranous VSD is more common in women over the age of 80, and in most of these cases, postdilatation with balloon catheter were performed Edwards Sapien and Corevalve drapes were used in the reported cases.^7-9^ In our case, a self-expanding portico valve was used. Postdilatation was performed during the procedure due to AR. During the procedure, as in our case, this important complication may be overlooked. Therefore, we think that more care should be taken, especially in cases whose calcifications have spread to LVOT and need to be postdilated.

 In these patients, in the literature, transcatheter closure is preferred for VSD closure because of the high surgical risk of these patients.^7-9^ In our case it was successfully closed with retrograde technique with a 10 mm muscular VSD occluder device.

## Conclusion

 In our case, the location of calcification could possibly caused VSD development. Although the frequency of VSD after post-TAVI is not known, there have been reported cases in patients with both Medtronic Corevalve and Edwards Sapien bioprosthetic valves implanted. This is the first reported VSD case complicating self-expandable Portico valve TAVI.

## Funding

 This study was not funded or supported by any organization.

## Ethical approval

 None.

## Competing interest

 All Authors declared that there is no conflict of interest.
